# Synthesis of Metastable Ternary Pd-W and Pd-Mo Transition Metal Carbide Nanomaterials

**DOI:** 10.3390/molecules26216650

**Published:** 2021-11-02

**Authors:** James M. Thode, Daniel P. Harris, Cheng Wan, Brian M. Leonard

**Affiliations:** Department of Chemistry, University of Wyoming, 1000 E. University Avenue, Laramie, WY 82071, USA; jthoderu@gmail.com (J.M.T.); dharri38@uwyo.edu (D.P.H.); cwan1@uwyo.edu (C.W.)

**Keywords:** carbide, palladium, catalysis, nanomaterials

## Abstract

Research and catalytic testing of platinum group transition metal carbides have been extremely limited due to a lack of reliable, simple synthetic approaches. Powder samples have been reported to phase separately above 1%, and only thin-film samples have been reported to have appreciable amounts of precious metal doping. Herein, we demonstrated, through the simple co-precipitation of Pd and W or Mo precursors and their subsequent annealing, the possibility to readily form ternary carbide powders. During the investigation of the Pd-W ternary system, we discovered a new hexagonal phase, (PdW)_2_C, which represents the first non-cubic Pd ternary carbide. Additionally, the solubility of Pd in the Pd-W-C and Pd-Mo-C systems was increased to 24 and 32%, respectively. As a potential application, these new materials show an enhanced activity for the methanol oxidation reaction (MOR) compared to industrial Pd/C.

## 1. Introduction

Platinum group metals and their compounds have a widespread use in both technological and industrial applications. Both Pt and Pd are highly active for various electrocatalytic processes including hydrogen evolution reaction (HER), oxygen reduction reaction (ORR), and methanol oxidation reaction (MOR) [1,2,3]. However, there are several limitations to Pt group catalysts like poisoning, aggregation, and stability in harsh environments [4]. Previous researchers have alloyed Pt and Pd with other metals to improve reactivity and stability, but there is still room for improvement. One area of research that has been particularly overlooked is Pt group metal carbides. This is primarily due to limited synthetic approaches leading to their formation. In fact, very few binary carbides exist for group 7–10 metals, and most of those reported phases have a limited stability in water and acid [5]. The heat of formation for these binary carbide compounds becomes less negative moving from left to right along the d-block, with TiC being the most stable carbide [6]. TiC has a ΔH^°^_f_ of −92.9 kJ/mole, while V_4_C_3_ increases to −40.3 kJ/mole. For late transition metal carbides like Fe_3_C, the heat of formation flips signs and goes positive with a value of 4.7 kJ/mole for Fe_3_C. Even though there is a limited stability with these compounds, they provide another avenue for electronic tuning because the metal-carbon bond strength decreases when the number of *d*-electrons increases in late transition metals, as confirmed by ab initio calculations [7,8].

Although no stable carbides exist for platinum group metals, it has been demonstrated that such phases do exist as metastable compounds [9]. For example, the formation of a palladium carbide-like phase has been demonstrated during the hydrogenation of 1-pentyne over a palladium catalyst [10]. In this system, the carbide phase is selective for alkene production while the hydride phase favors alkanes. Further evidence of the formation of surface PdC has been seen during the catalytic combustion of lean methane/air mixtures over a Pd catalyst, during dehydrogenation reactions, and during methane production [11,12,13,14,15]. Perhaps the most important discovery came from pioneering work by Ono et al. [16]. They demonstrated the synthesis of cubic PtC in a laser-heated diamond anvil cell by heating platinum powder and carbon to 2300 °C at a pressure of 85 GPa. This resulted in several investigations into the mechanical properties of this material using first principle calculations [17,18,19,20,21,22,23,24,25,26,27]. Most recently, PdC was also reported from a diamond anvil synthesis at 50 GPa and 2500 KN [28].

A more promising approach to Pt group carbides exists with ternary (or pseudo binary) compounds, although most examples show a very low solubility or limited evidence of incorporation of the second metal. In 2004, it was shown that low levels of transition metals could be used to selectively form either α-MoC_1−x_ (cubic) or β-Mo_2_C (hexagonal) [29]. Pd was added at 0.2% to produce the cubic phase of molybdenum carbide as a powder, with no metallic Pd observed by X-ray diffraction (XRD). Likewise, 0.25% Pt was added, forming a similar rock-salt carbide phase. In 2014, Pt-doped molybdenum carbides were investigated for the low temperature steam reforming of methanol [30]. This investigation revealed that Pt metal phase separation occurred above 1.2%. The most success with respect to Pd and Pt solubility was reported in thin film synthesis via sputter deposition. Gregoire et al. demonstrated a solid solution for the entire composition range of Pd_x_W_1−x_C and Pt_x_W_1−x_C [31].

Herein, we report a synthetic approach capable of incorporating large amounts of Pd in a powder carbide sample (7–32%), far exceeding any previously published results. Pd insertion within the carbide lattice is demonstrated using XRD, scanning electron microscopy (SEM), and transmission electron microscopy (TEM). We also discovered a new hexagonal phase, (Pd_x_W_1−x_)_2_C, the first example of Pd solubility in a noncubic carbide structure.

## 2. Results and Discussion

It is valuable, for greater clarity, to briefly discuss the various phases of molybdenum and tungsten carbide present within this article. Both the Mo-C and W-C systems have phases with MC and M_2_C stoichiometry. α-MoC_1−x_ and γ-WC_1−x_ have a face-centered cubic structure (fcc) with ABCABC stacking. β-Mo_2_C and W_2_C are in a hexagonal close-packed arrangement (hcp) with ABAB stacking. In the case of W-C, there is also an α-WC phase (also known as WC type) with a simple hexagonal structure and AA stacking [32]. While other phases of Mo-C do exist, they are not pertinent to this publication [33]. All of the crystal information can be found in Tables S1 and S2 of the Supporting Information. The following compounds, Pd_x_W_1−x_C, (Pd_x_W_1−x_)_2_C, and Pd_x_Mo_1−x_C, were all made using the amine metal oxide composite method. A detailed description of the synthesis can be found in Section 3. In short, precursors of Pd (K_2_PdCl_6_), W (ammonium metatungstate) or Mo (ammonium heptamolybdate), and C (*o*-phenylenediamine) were added to an aqueous solution while stirring at 45 °C. The mixture was then co-precipitated by careful addition of 3 M HCl to a pH below 3. The precipitate was then annealed at a variety of temperatures to study the composition and phase formation.

**Figure 1:**
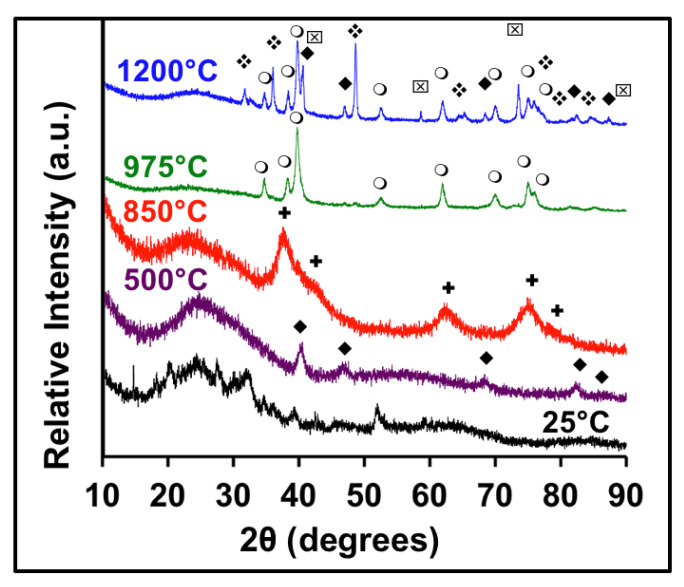
X-ray diffraction (XRD) of Pd and W ternary carbides. 25 °C (black), 500 °C (purple), 850 °C (red), 975 °C (green), 1200 °C (blue). The symbols in the figure are based on the following JCPDS files ♦ = Pd metal 46-1043, ☒ = W metal 00-004-0806, ✚ = PdWC, ❍ = (PdW)_2_C, ❖ = WC-hex 00-025-1047).

Figure 1 shows XRD data demonstrating the presence of Pd_x_W_1−x_C and (Pd_x_W_1−x_)_2_C. This sample was made by combining a 2:1 ratio of W and Pd. The XRD data reveals a number of transitions starting with the AMOC precursor at room temperature, whose diffraction pattern does not match any known patterns in the PDF database. At 500 °C, metallic Pd peaks first emerge, followed by Pd_x_W_1−x_C at 850 °C, which forms in the cubic γ-WC_1−x_ structure. At 850 °C, the Pd peaks are no longer present, and the diffraction peaks have all shifted to lower angles, with the formation of the carbide indicating that the Pd has dissolved into the lattice of the WC phase. The lattice constants for the synthesized materials and peak positions are listed in Table S2. The lattice constant for the cubic Pd-W-C phase is 4.252 Å, which correlates well with Gregoire et al. who reported ~4.2 Å for compounds with 0–25% Pd and with other reports of WC and WC_1−x_ [31]. At 975 °C, a unique diffraction pattern is observed, which can be indexed to a hexagonal unit cell with a similar structure to the β-W_2_C phase. This compound, (Pd_x_W_1−x_)_2_C, has never been cited in the literature and is the first synthetic example of Pd bound to carbon in a noncubic structure [34,35]. All previous publications of Pd in WC and MoC showed the rock-salt phase as the preferred crystal structure, including both powder and thin-film samples. However, with this synthetic approach, the hexagonal structure of (PdW)_2_C can be isolated as a stable phase. Finally, at 1200 °C, the metastable ternary carbide phase separates into hexagonal δ-WC and metallic Pd, along with remnants of the β-W_2_C phase. This series of data helps identify the location of Pd during the various transition temperatures. Pd is reduced to a metallic state from the precursor material at low temperatures, dissolves into the carbide lattice in two phases of tungsten carbide, and finally separates into an elemental state again at high temperatures, where the δ-WC phase also appears.

The XRD data can be correlated with the thermogravimetric analysis (TGA) data from Figure S1, which is similar to previous studies on carbide formation [36]. From 25–225 °C, there is a mass loss that corresponds to the loss of water and volatile species present in the precursor. From 225–300 °C, another event occurs as free amine leaves the system. From 300–425 °C, there is another mass loss, which gives rise to the formation of crystalline Pd, as seen in the XRD from Figure 1. This mass loss is likely due to the decomposition of palladium chloride into Pd metal and HCl gas. Finally, the mass loss at 800 °C corresponds to the reduction of W and subsequent formation of carbide. While this synthesis method is still under investigation, it was observed that the acidity of the reaction mixture had a significant effect on the solubility of Pd. As seen in Figure S2, pHs below the pKa_1_ of *o*-phenylenediamine, 0.80, result in metallic Pd being present as a distinct phase in the XRD. Phase pure ternary carbides were only obtained at pHs above 0.8.

**Figure 2:**
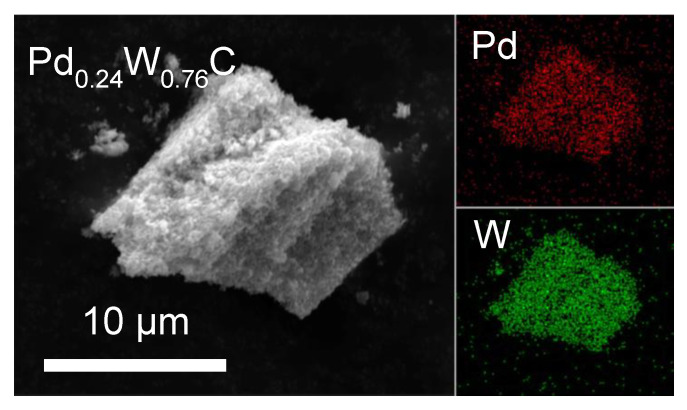
SEM of Pd-W cubic ternary carbide annealed to 850 °C with 24% Pd. The elemental mapping of these particles shows uniform Pd (red) and W (green).

To further demonstrate the inclusion of Pd into the WC lattice, SEM images with energy dispersive X-ray spectroscopy (EDS) mapping were studied. Figure 2 shows the cubic Pd-W-C ternary carbide with 24% Pd. Other compositions including 15% and 7% were also synthesized, with the NaCl structure indicating a significant degree of composition control. The hexagonal carbide was found at 15% and 24% Pd. The EDS mapping shows homogeneity with both Pd and W found in the same locations and compositions throughout the samples, indicating a uniform distribution within the carbide. Figure S3 shows the XRD patterns and SEM/EDS for samples with varying compositions. Table S3 shows the % Pd content based on EDS measurements, further confirming composition control in these phases. There is no interference between the Pd and W peaks in the EDS spectra, allowing for a confident analysis of their compositions (see Figure S4).

**Figure 3:**
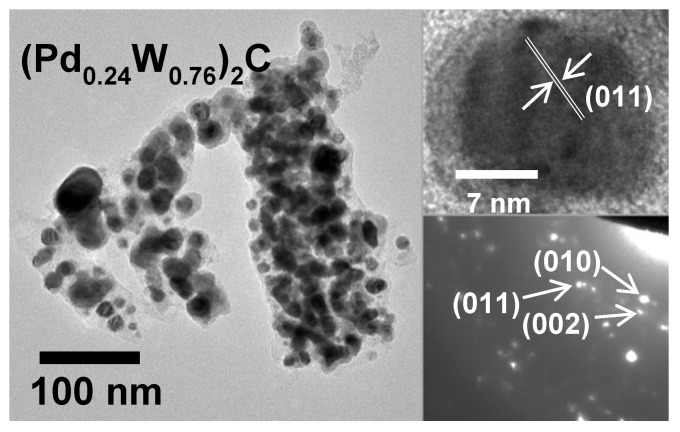
Bright-field TEM image of Pd-W hexagonal ternary carbide annealed to 975 °C with 24% Pd. The lattice fringe analysis and SAED further confirm the hexagonal structure.

The particle size, lattice constant measurements, and selected area electron diffraction (SAED) were analyzed using high-resolution TEM, as shown in Figure 3. The particles appear to be made of 5–20 nm spheres that aggregate to form roughly 100 nm clusters. Lattice fringes were measured to be 0.22 nm, in close agreement with the (011) plane of (PdW)_2_C. SAED in Figure 3 also confirms the presence of (PdW)_2_C. EDS from the TEM samples demonstrates the homogeneous presence of both Pd and W on the nanometer scale, as seen in Figure S5.

**Figure 4:**
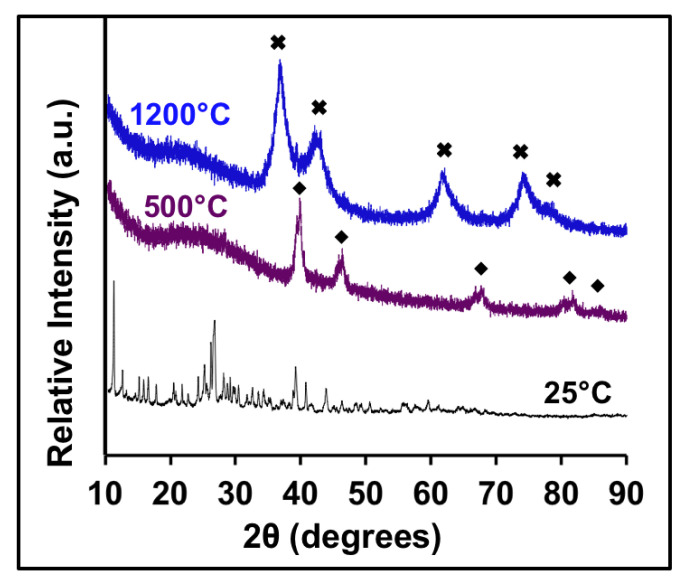
XRD of Pd and Mo ternary carbides at 25 °C (black), 500 °C (purple), and 1200 °C (blue). The symbols for (♦) Pd in the figure are based on JCPDS files Pd46-1043, and PdMoC (✖) does not have a reported structure.

Molybdenum carbide was also reported to have a preferential phase growth with 0.2% Pd doping, causing the material to adopt the cubic phase MoC rather than the common hexagonal Mo_2_C. In the present investigation, rock-salt MoC was also found, but with Pd doping now reaching 32%. From the TGA data in Figure S1, free amine was lost at 300 °C followed by a Pd reduction at ~400 °C. However, carbide formation does not occur until 1000 °C or higher for the Mo carbide system. At 500 °C, the XRD reveals peaks belonging to Pd metal, as seen in Figure 4. The peaks shift to lower angles at 1200 °C as the lattice constant increases to 4.240 Å, indicating the formation of the rock-salt Pd_x_Mo_1−x_C. No Pd peaks can be observed at higher temperatures, indicating the full dissolution of the metallic Pd into the Mo carbide lattice.

**Figure 5:**
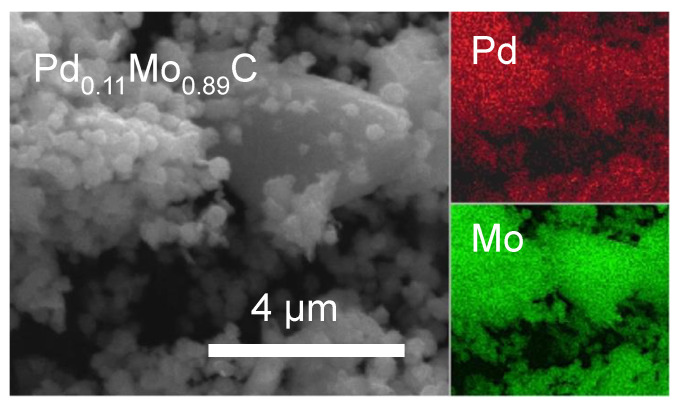
SEM of Pd-Mo cubic ternary carbide annealed to 1200 °C with 11% Pd. The elemental mapping of the sample shows uniform Pd (red) and Mo (green).

The SEM of Pd_0.11_Mo_0.89_C annealed to 1200 °C is shown in Figure 5. The EDS mapping shows the carbide to be homogenous, with both Pd and Mo present in the same locations of the SEM images, again indicating a uniform elemental dispersion within the carbide. Table S3 shows the % Pd content based on EDS measurements, demonstrating a good composition control including 32%, 22%, and 11% Pd. The particles appear mostly spherical in the SEM, with some aggregates appearing slightly plate-like.

**Figure 6:**
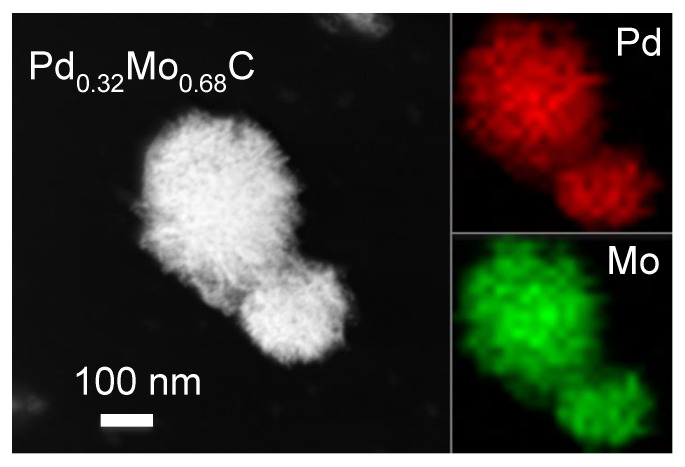
Dark-field TEM image of Pd-Mo cubic ternary carbide annealed to 1200 °C with 32% Pd. The elemental mapping of the particles shows uniform Pd (red) and Mo (green).

Figure 6 shows a dark-field TEM image of PdMoC with fibrous particles aggregating into 150 nm clusters. The EDS mapping confirms the existence of both Pd and Mo at the nm scale in the TEM image. There is, again, a lack of interference between the Pd and Mo peaks in the EDS spectrum (see Figure S6). The TEM images shown in Figure S7 reveal spherical particles with diameters of 7–12 nm. The observed lattice fringes have a spacing of 0.26 nm, in close agreement with the (111) plane of PdMoC. SAED also confirms the cubic structure of PdMoC.

**Figure 7:**
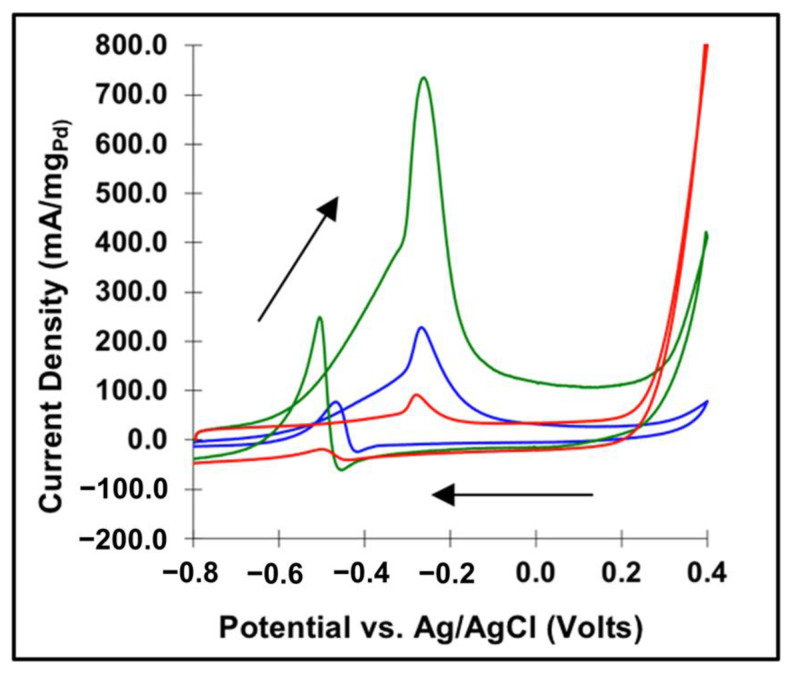
Cyclic voltammogram of 9.5% (PdW)_2_C (green), 5.5% PdMoC (blue), and 10.5% Pd/C (red) in 1 M MeOH/1 M KOH.

To investigate these materials’ potential application as electrocatalysts, their activity for the methanol oxidation reaction was tested. Figure 7 displays the cyclic voltammetry of 9.5% PdW_2_C (green), 5.5% PdMoC (blue), and 10.5% Pd@C (red) in a solution of 1.0 M methanol in 1.0 M KOH. The peak current values for the anodic sweep are: 734.5 mA/mg_Pd_ for (Pd_0.095_W_0.905_)_2_C, 228.1 mA/mg_Pd_ for Pd_0.055_Mo_0.945_C, and 91.1 mA/mg_Pd_ for 10.5% Pd@C. These loadings were verified using ICP-OES, as seen in Table S5. This increase in electrochemical activity can be attributed to the favorable interaction between Pd in a carbide lattice as compared to when it is deposited on a carbon support. In addition, the X-ray Photoelectron Spectroscopy (XPS) of these samples showed that the Pd 3d peaks had shifted to 335.9 eV, similar to previous reports of Pd-C, as seen in the supporting info in Figures S8 and S9 [10]. Carbides have in general been shown to be superior catalysts for a variety of reactions including HER, ORR, and MOR, and the addition of Pd further enhanced their activity [37].

## 3. Materials and Methods

Pd-W-C synthesis. In a typical experiment, a ratio of 1:25 (moles of metal:moles of carbon) is weighed out. For a sample of 2.85:1 (W:Pd), this results in 0.0623 g K_2_PdCl_6_ being weighed out in a 20 mL scintillation vial and dissolved in 15 mL D.I. H_2_O. Its contents are sonicated for 30 min in an ultrasonicator. In a separate 100 mL beaker, 0.0710 g of *o*-phenylenediamine are weighed out in addition to 0.1965 g of ammonium metatungstate. The contents of the beaker are dissolved in 60 mL of D.I. water and placed on a hot plate at 45 °C while stirring for 20 min. After the K_2_PdCl_6_ has finished sonicating, its contents are added to the reaction beaker. From here, a pH probe is used to monitor the addition of 1:3 conc. HCl:D.I. H_2_O. The pH is subsequently dropped to just below 3. The sample is stirred vigorously at 45 °C for 2 h. At this time, a precipitate will have formed. The reaction is then cooled to room temperature. Stirring is turned off, and the reaction beaker is placed on a lab bench for 10 min or until all of the precipitate has sunk to the bottom. From here, the supernatant is decanted off into a waste container, the stir bar is removed, and the remaining contents are placed in a drying oven at 60 °C to dry for 1 h. The precursor powder is then collected and taken to an X-ray diffractometer (Rigaku Smart Lab) for data collection. Finally, the sample is ready for annealing in a thermogravimetric analyzer (model: TA Instruments SDT Q600) under an Ar flow of 100 mL/min to the desired reaction temperature at a ramping rate of 20 °C/min.

Pd-Mo-C synthesis. In a typical experiment, a ratio of 1:20 (moles of metal:moles of carbon) is weighed out. For a sample of 2.27:1 (Mo: Pd), this results in 0.0397 g K_2_PdCl_6_ being weighed out in a 20 mL scintillation vial and dissolved in 15 mL D.I. H_2_O. Its contents are sonicated for 30 min in an ultrasonicator. In a separate 100 mL beaker, 0.1151 g of *o*-phenylenediamine are weighed out in addition to 0.0377 g of ammonium heptamolybdate. The contents of the beaker are dissolved in 60 mL of D.I. water and placed on a hot plate at 45 °C while stirring for 20 min. After the K_2_PdCl_6_ has finished sonicating, its contents are added to the reaction beaker. From here, a pH probe is used to monitor the addition of 1:3 conc. HCl:D.I. H_2_O. The pH is subsequently dropped to just below 3. The sample is stirred vigorously at 45 °C for 2 h. At this time, a precipitate will have formed. The reaction is then cooled to room temperature. Stirring is turned off, and the reaction beaker is placed on a lab bench for 10 min or until all of the precipitate has sunk to the bottom. From here, the supernatant is decanted off into a waste container, the stir bar is removed, and the remaining contents are placed in a drying oven at 60 °C to dry for 1 hr. The precursor powder is then collected and taken to an X-ray diffractometer for data collection. Finally, the sample is ready for TGA annealing under an Ar flow of 100 mL/min to the desired reaction temperature at a ramping rate of 20 °C/min. All samples were rapidly cooled to room temperature.

### 3.1. TEM Grid Preparation

TEM grids were prepped with ~1 mg of sample sonicated in 3 mL of ethanol for 45 min. The dispersed sample, light grey in color, was then drop-cast (0.2 mL) onto a Cu or Ni grid. In order to reduce drift under TEM, the sample was allowed to dry for 12 h before being analyzed.

### 3.2. X-ray Photoelectron Spectroscopy

Measurements were performed on a Physical Electronics 5800 instrument. High resolution spectra were shifted to a C1s hydro carbon peak at 284.8 eV. High resolution spectra were collected at a pass energy of 23.5 eV in 0.1 eV increments. A low energy electron gun was used to neutralize any charging.

### 3.3. Electrochemical Analysis

The GCEs are cleaned prior to the drop cast procedure via polishing with 5 μm alumina powder to produce a shiny and clear surface, then sonicated for 5 min in D.I. water, and finally electrochemically cleaned with 5 CVs in 1.0 M KOH. Catalysts were prepared by the addition of 5 mg powder to 625 μL D.I. H_2_O and 125 μL nafion. The mixture was sonicated for 1 h to produce a homogenous, black ink. 3 μL of the catalyst ink was immediately drop-cast onto a 3 mm glassy carbon electrode (GCE). The electrodes were allowed to dry for 12 h before use. Once the electrodes were prepared, 50 CVs were performed in 1.0 M MeOH/1.0 M KOH. The 50th scan has been reported for comparison.

## 4. Conclusions

In conclusion, this low temperature synthesis route was shown to greatly enhance the solubility of Pd in both Mo-C and W-C carbide systems. Because all three elements are coordinated in the precursors, they combine to form ternary (or pseudo-binary) metal carbide nanomaterials at low temperatures. Through this investigation, a new compound, (PdW)_2_C, was synthesized. Furthermore, the solubility of Pd in these Pd-W-C and Pd-Mo-C systems was greatly increased to 24 and 32%, respectively. The composition control was demonstrated for both systems, which allows yet another avenue for tuning the catalytic activity. Finally, these materials showed an enhanced catalytic activity for methanol oxidation and will likely show a catalytic activity for many other processes. Given the promise of Gregoire’s solid solutions in thin films, it is possible that the solubility can be further increased, and it is likely that other late transition metals will find an increased solubility in these metal carbide systems.

## Data Availability

The data presented in this study are available on request from the corresponding author. The data are not publicly available due to pending patent applications.

## Supplementary Materials

The following are available online. Figure S1: Thermogravimetric analysis (TGA) of PdMoC (blue) and PdWC/PdW_2_C (red) with a 2:1 metal:Pd ratio. Figure S2: (a) XRD of PdWC synthesized at 850 °C (pH = 0.5 (bottom) and pH = 2.65 (top)) Pd (♦), PdWC (✚), and (PdW)_2_C (❍). (b) XRD of PdMoC synthesized at 1000 °C (pH = 0.5 (bottom) and pH = 2.41 (top)) Pd (♦), PdMoC (✖) and Mo (☒). Figure S3: Variable loading of PdWC, (PdW)_2_C, and PdMoC. XRD shows different amounts (%) of Pd found in the sample by EDS via SEM. Figure S4: Example of EDS spectra of Pd_x_W_1−x_C. Figure S5: Dark-field STEM and corresponding EDS mapping for (PdW)_2_C. Figure S6: Example of EDS spectra of Pd_x_Mo_1−x_C. Figure S7: Bright-field TEM image of Pd-Mo cubic ternary carbide (32% addition) with lattice fringe analysis and SAED. Figure S8: XPS Data for PdWC with (a) survey scan, (b) W4f region, and (c) Pd3d region. Figure S9: XPS Data for (PdW)2C with (a) survey scan, (b) W4f region, and (c) Pd3d region. Table S1: Structural information of Pd-M-Cs with literature comparisons. Table S2: Lattice constants and peak positions for carbides with and without Pd insertion. Table S3: W loadings measured before and after synthesis with corresponding pH, temp., and resulting phase. Table S4: Mo loadings measured before and after synthesis with corresponding pH, temp., and resulting phase. Table S5: ICP-OES of 10.5% Pd/C, 9.5% (PdW)_2_C, and 5.5% PdMoC for electrochemical analysis.

## Sample Availability

Samples of the compounds are not currently available.

## References

[1] Li F., Ciani I., Bertoncello P., Unwin P.R., Zhao J., Bradbury C.R., Fermin D.J. (2008). Scanning Electrochemical Microscopy of Redox-Mediated Hydrogen Evolution Catalyzed by Two-Dimensional Assemblies of Palladium Nanoparticles. J. Phys. Chem. C.

[2] Ma X., Meng H., Cai M., Shen P.K. (2012). Bimetallic Carbide Nanocomposite Enhanced Pt Catalyst with High Activity and Stability for the Oxygen Reduction Reaction. J. Am. Chem. Soc..

[3] Bianchini C., Shen P.K. (2009). Palladium-Based Electrocatalysts for Alcohol Oxidation in Half Cells and in Direct Alcohol Fuel Cells. Chem. Rev..

[4] Borup R., Meyers J., Pivovar B., Kim Y.S., Mukundan R., Garland N., Myers D., Wilson M., Garzon F., Wood D. (2007). Scientific aspects of polymer electrolyte fuel cell durability and degradation. Chem. Rev..

[5] Liu Y., Kelly T.G., Chen J.G., Mustain W.E. (2013). Metal carbides as alternative electrocatalyst supports. ACS Catal..

[6] Zhang Y., Li J., Zhou L., Xiang S. (2002). A theoretical study on the chemical bonding of 3d-transition-metal carbides. Solid State Commun..

[7] Häglund J., Guillermet A.F., Grimvall G., Körling M. (1993). Theory of bonding in transition-metal carbides and nitrides. Phys. Rev. B.

[8] Meschel S.V., Kleppa O.J. (1997). Standard enthalpies of formation of some 3d transition metal carbides by high temperature reaction calorimetry. J. Alloys Compd..

[9] Ziemecki S.B., Jones G.A., Swartzfager D.G., Harlow R.L., Faber J. (2002). Formation of interstitial palladium-carbon phase by interaction of ethylene, acetylene, and carbon monoxide with palladium. J. Am. Chem. Soc..

[10] Teschner D., Borsodi J., Wootsch A., Révay Z., Hävecker M., Knop-Gericke A., Jackson S.D., Schlögl R. (2008). The roles of subsurface carbon and hydrogen in palladium-catalyzed alkyne hydrogenation. Science.

[11] Setiawan A., Kennedy E.M., Dlugogorski B.Z., Adesina A.A., Tkachenko O., Stockenhuber M. (2014). Evidence of the Formation of Surface Palladium Carbide during the Catalytic Combustion of Lean Methane/Air Mixtures. Energy Technol..

[12] Schuster R., Bertram M., Runge H., Geile S., Chung S., Vonk V., Noei H., Poulain A., Lykhach Y., Stierle A. (2021). Metastability of palladium carbide nanoparticles during hydrogen release from liquid organic hydrogen carriers. Phys. Chem. Chem. Phys..

[13] Guo R., Chen Q., Li X., Liu Y., Wang C., Bi W., Zhao C., Guo Y., Jin M. (2019). PdC x nanocrystals with tunable compositions for alkyne semihydrogenation. J. Mater. Chem. A.

[14] Wang C., Jia Y., Zhang Z., Zhao G., Liu Y., Lu Y. (2019). Role of PdC x species in Pd@PdC x /AlOOH/Al-fiber catalyst for the CO oxidative coupling to dimethyl oxalate. Appl. Surf. Sci..

[15] Jones W., Wells P.P., Gibson E.K., Chutia A., Silverwood I.P., Catlow C.R.A., Bowker M. (2019). Carbidisation of Pd Nanoparticles by Ethene Decomposition with Methane Production. ChemCatChem.

[16] Ono S., Kikegawa T., Ohishi Y. (2005). A high-pressure and high-temperature synthesis of platinum carbide. Solid State Commun..

[17] Li L., Yu W., Jin C. (2005). First-principles calculations of a high-pressure synthesized compound PtC. J. Phys. Condens. Matter.

[18] Fan C.-Z., Zeng S.-Y., Zhan Z.-J., Liu R.-P., Wang W.-K., Zhang P., Yao Y.-G. (2006). Low compressible noble metal carbides with rocksalt structure: Ab initio total energy calculations of the elastic stability. Appl. Phys. Lett..

[19] Demirtas M., Ustunel H., Toffoli D. (2017). Effect of Platinum, Gold, and Potassium Additives on the Surface Chemistry of CdI2-Antitype Mo2C. ACS Omega.

[20] Fan C.Z., Sun L.L., Wang Y.X., Liu R.P., Zeng S.Y., Wang W.K. (2006). First-principles study on the structural, elastic and electronic properties of platinum carbide. Phys. B Phys. Condens. Matter.

[21] Rabah M., Rached D., Ameri M., Khenata R., Zenati A., Moulay N. (2008). Theoretical study of ground state and high-pressure phase of platinum carbide. J. Phys. Chem. Solids.

[22] Peng F., Fu H.Z., Yang X.D. (2008). Transition phase and thermodynamic properties of PtC from first-principles calculations. Solid State Commun..

[23] Deligoz E., Ciftci Y.O., Jochym P.T., Colakoglu K. (2008). The first principles study on PtC compound. Mater. Chem. Phys..

[24] Bannikov V.V., Shein I.R., Ivanovskii A.L. (2010). Trends in stability, elastic and electronic properties of cubic Rh, Ir, Pd and Pt carbides depending on carbon content: MC versus M4C from first-principles calculations. J. Phys. Chem. Solids.

[25] Mankad V., Rathod N., Gupta S.D., Gupta S.K., Jha P.K. (2011). Stable structure of platinum carbides: A first principles investigation on the structure, elastic, electronic and phonon properties. Mater. Chem. Phys..

[26] Zaoui A., Ferhat M. (2011). Dynamical stability and high pressure phases of platinum carbide. Solid State Commun..

[27] Li Q., Zhang X., Liu H., Wang H., Zhang M., Li Q., Ma Y. (2014). Structural and Mechanical Properties of Platinum Carbide. Inorg. Chem..

[28] Fedotenko T., Dubrovinsky L., Khandarkhaeva S., Chariton S., Koemets E., Koemets I., Hanfland M., Dubrovinskaia N. (2020). Synthesis of palladium carbides and palladium hydride in laser heated diamond anvil cells. J. Alloys Compd..

[29] Jung K.T., Kim W.B., Rhee C.H., Lee J.S. (2003). Effects of Transition Metal Addition on the Solid-State Transformation of Molybdenum Trioxide to Molybdenum Carbides. Chem. Mater..

[30] Ma Y., Guan G., Shi C., Zhu A., Hao X., Wang Z., Kusakabe K., Abudula A. (2014). Low-temperature steam reforming of methanol to produce hydrogen over various metal-doped molybdenum carbide catalysts. Int. J. Hydrog. Energy.

[31] Gregoire J.M., Tague M.E., Smith E.H., Dale D., DiSalvo F.J., Abruña H.D., Hennig R.G., van Dover R.B. (2010). Phase Behavior of Pseudobinary Precious Metal−Carbide Systems. J. Phys. Chem. C.

[32] Kurlov A.S., Gusev A.I. (2006). Tungsten carbides and W-C phase diagram. Inorg. Mater..

[33] Wan C., Regmi Y.N., Leonard B.M. (2014). Multiple phases of molybdenum carbide as electrocatalysts for the hydrogen evolution reaction. Angew. Chemie—Int. Ed..

[34] Wang B., Wang H.Q., Li Y.Q., Zhang S.Y., Hou J.G. (2000). Formation of a palladium carbide with a palladium-silicide-like structure in fullerene-C60/Pd multilayers. Mater. Res. Bull..

[35] Rabah M., Benalia S., Rached D., Abidri B., Rached H., Vergoten G. (2010). Prediction of stabilities phase and elastic properties of Palladium Carbide. Comput. Mater. Sci..

[36] Wan C., Knight N.A., Leonard B.M. (2013). Crystal structure and morphology control of molybdenum carbide nanomaterials synthesized from an amine-metal oxide composite. Chem. Commun..

[37] Regmi Y.N., Waetzig G.R., Duffee K.D., Schmuecker S.M., Thode J.M., Leonard B.M. (2015). Carbides of group IVA, VA and VIA transition metals as alternative HER and ORR catalysts and support materials. J. Mater. Chem. A.

