# Supplementary material for: Synthesis of Metastable Ternary Pd-W and Pd-Mo Transition Metal Carbide Nanomaterials
Source: Molecules. 2021 Nov 2;26(21):6650. doi: 10.3390/molecules26216650 (PMC8588312; doi:10.3390/molecules26216650)
Supplement: Supplementary file 1 [file molecules-26-06650-s001.zip › molecules-1426527-supplementary.pdf]

Supporting Information for

Synthesis of metastable ternary Pd-W and Pd-Mo transition metal carbide nanomaterials

James M. Thode[1], Daniel P. Harris[1], Cheng Wan[1], and Brian M. Leonard[1,*]

Table S1. Structural information of Pd-M-Cs with literature comparisons.

Structure/Crystal view	Phase	Maximum Pd loading (atomic % based on Mo or W)	Sample type	Author
**$Pd_xW_{1-x}C$:** 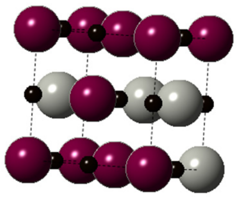	$Pd_xW_{1-x}C$	82.5	thin film	Gregoire et al.
	$Pd_xW_{1-x}C$	24	powder	Thode et al.
**$(Pd_xW_{1-x})_2C$:** 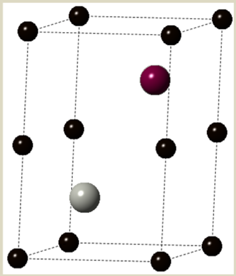	$(Pd_xW_{1-x})_2C$	24	powder	Thode et al.
**$Pd_xMo_{1-x}C$:** 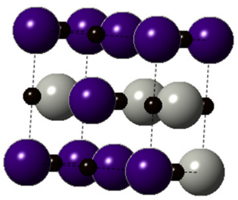	$Pd_xMo_{1-x}C$	0.12 (0.2% by mass)	powder	Jung et al.
	$Pd_xMo_{1-x}C$	0.12	powder	Thode et al.

Table S2. Lattice constants and peak positions for carbides with and without Pd insertion.

Compound	Lattice Constant	Peak Positions
MoC_{1-x}***	4.270 Å	36.6°, 42.4°, 61.5°, 73.6°, 77.5°
$Pd_xMo_{1-x}C_x$	4.240 Å	36.8°, 42.8°, 62.0°, 74.2°, 78.1°
WC ***	4.266 Å	36.4°, 42.3°, 61.4°, 73.6°, 77.4°
$WC_{0.85}$***	4.252 Å	36.6°, 42.5°, 61.6°, 73.9°, 77.7° (instrumental shift of + 0.71 degrees)
$Pd_xW_{1-x}C$	4.258 Å	37.2°, 43.1°, 62.3°, 74.5°, 78.3°
W_2C ***	a/b= 2.985 Å c= 4.717 Å	34.7°, 38.1°, 39.7°, 52.5°, 62.1°, 70.0°, 73.1°, 75.3°, 76.3°, 81.6°, 85.6°
$(Pd_xW_{1-x})_2C$	a/b= 3.013 Å c= 4.750 Å	34.3°, 37.9°, 39.3°, 52.0°, 61.5°, 69.4°, 72.4°, 74.5°, 75.5°, 80.9° 84.7°

***Lattice constant for MoC_{1-x} (PDF card number 01-098-2868), WC, $WC_{0.85}$, and W_2C (SI reference 1)

Table S3. W loadings measured before and after synthesis with corresponding pH, temp., and resulting phase.

W:Pd molar ratio measured "in" precursor synthesis	W:Pd molar ratio "out" by EDS	pH	Temp. (20°C/ min ramp under Ar flow)	Phase type
2.85:1 (35%)	24%	2.87	850°C	cubic
2.85:1 (35%)	24%	2.87	975°C	hexagonal
6.89:1 (14.5%)	15%	2.80	850°C	cubic
6.89:1 (14.5%)	15%	2.80	975°C	hexagonal
16.2:1 (5.9%)	7%	1.27	1000°C	cubic

Table S4. Mo loadings measured before and after synthesis with corresponding pH, temp. and resulting phase.

W:Pd molar ratio measured "in" precursor synthesis	W:Pd molar ratio "out" by EDS	pH	Temp. (20°C/ min ramp under Ar flow)	Phase type
2.27:1 (33%)	32%	2.40	1000°C	
3.16:1 (24%)	22%	1.90	1200°C	cubic
17.25:1 (5.5%)	11%	2.04	1000°C	

Table S5. ICP-OES of 10.5% Pd/C, 9.5% $(PdW)_2C$, and 5.5% PdMoC for electrochemical analysis.

**Metal:Pd molar ratio measured "in" precursor synthesis**	**Metal:Pd molar ratio "out" by EDS**	**% Pd by ICP-OES**	**pH**	**Temp. (20°C/ min ramp under Ar flow)**	**Phase type**
10% Pt/C	/	10.5%	/	/	/
6.6:1, W:Pd (13%)	12%	9.5%	2.79	975°C	$(PdW)_2C$
19.75:1, (4.8%)	7%	5.5%	2.37	1000°C	PdMoC

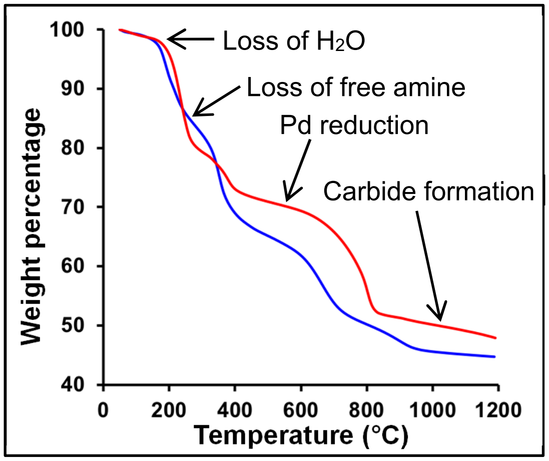

Figure S1. Thermogravimetric analysis (TGA) of PdMoC (blue) and PdWC/PdW_2C (red) with a 2:1 metal:Pd ratio.

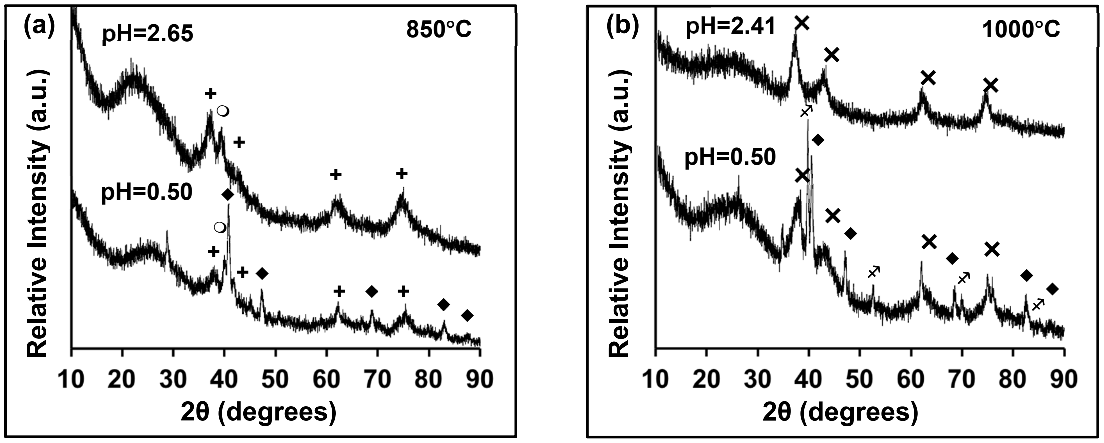

Figure S2. (a) XRD of PdWC synthesized at 850°C (pH=0.5 (bottom) and pH=2.65 (top)) Pd (◎), PdWC (✚), & $(PdW)_2C$ (◎). **(b)** XRD of PdMoC synthesized at 1000°C (pH=0.5 (bottom) and pH=2.41 (top)) Pd (◎), PdMoC (✖) & Mo (◎).

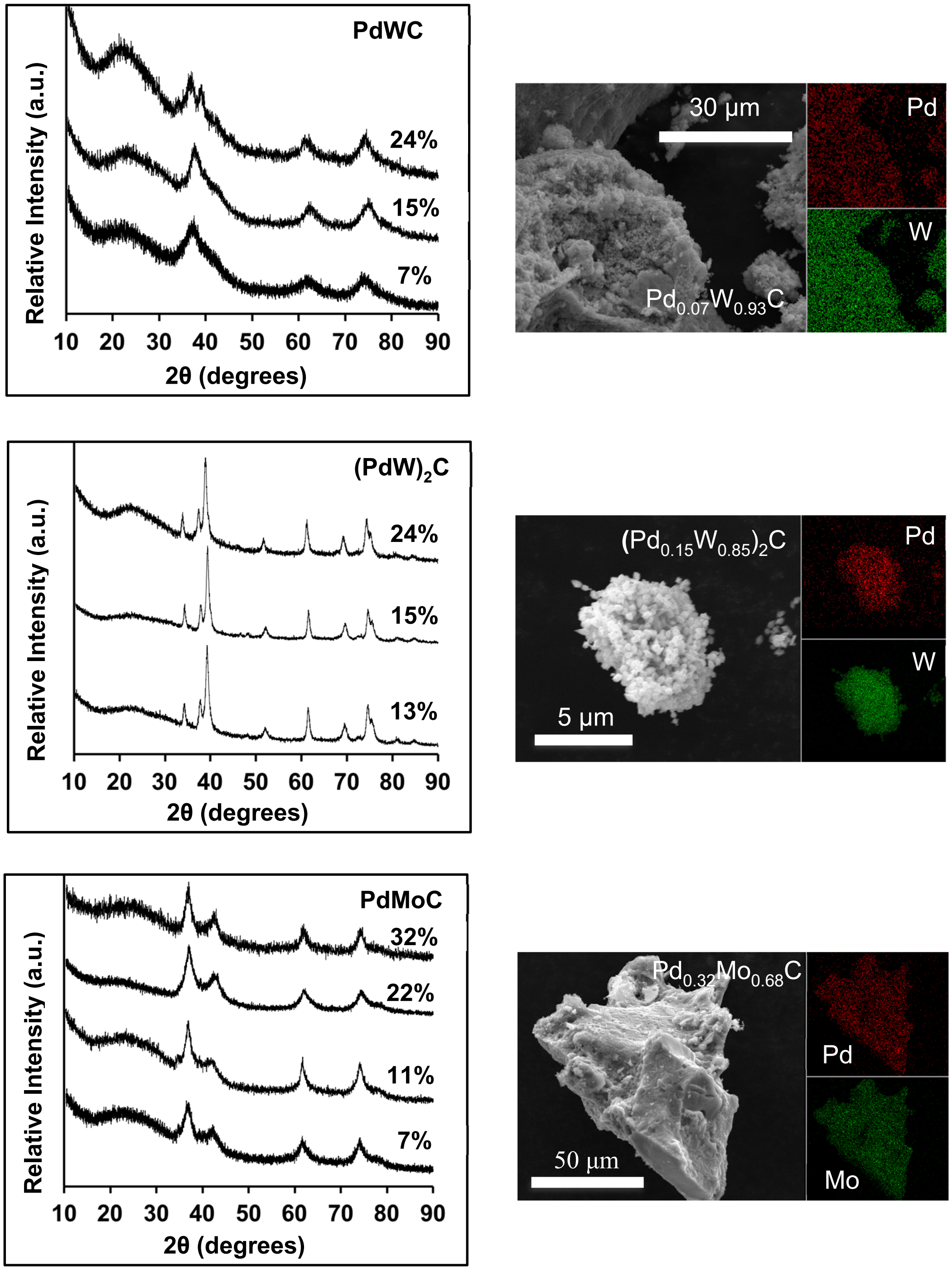

Figure S3. Variable loading of PdWC, $(PdW)_2C$, and PdMoC. XRD shows different amount (%) of Pd found in the sample by EDS via SEM.

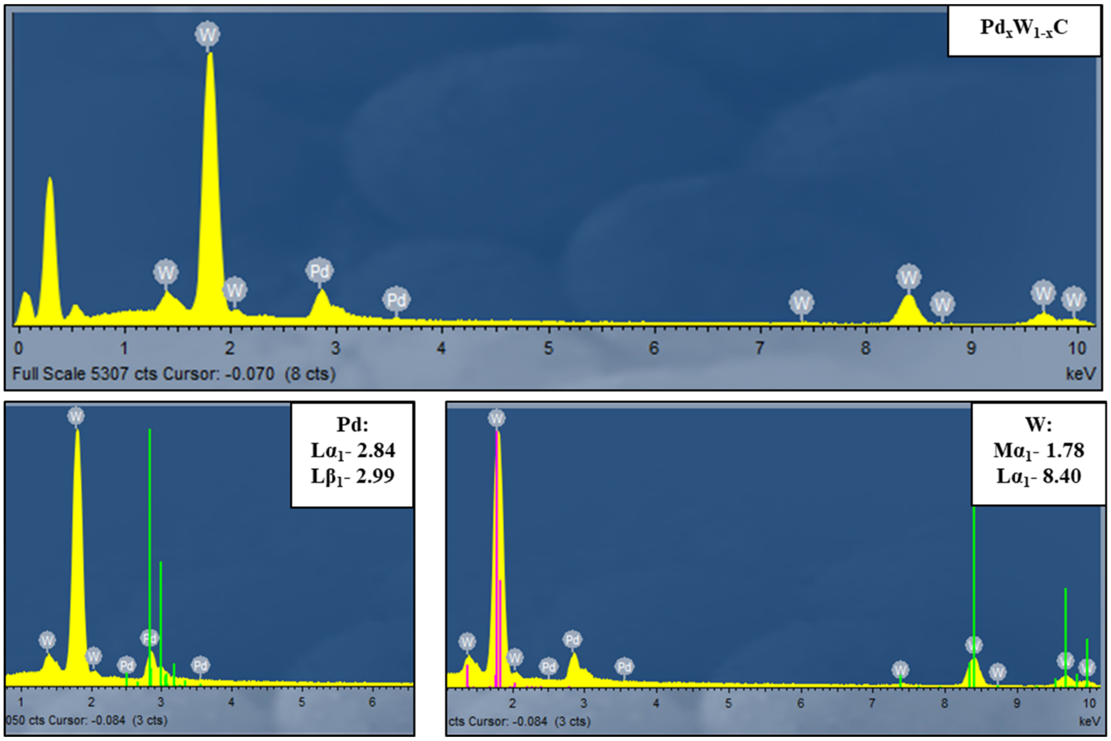

Figure S4. Example of EDS spectra of $Pd_xW_{1-x}C$.

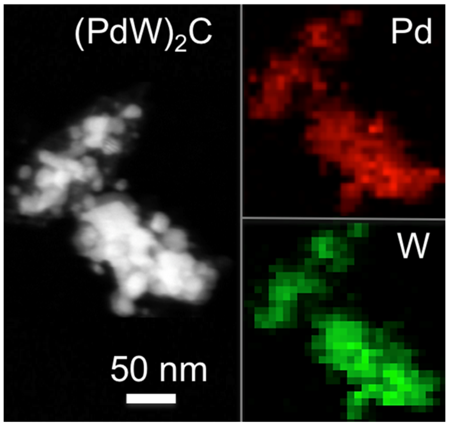

Figure S5. Darkfield STEM and corresponding EDS mapping for $(PdW)_2C$.

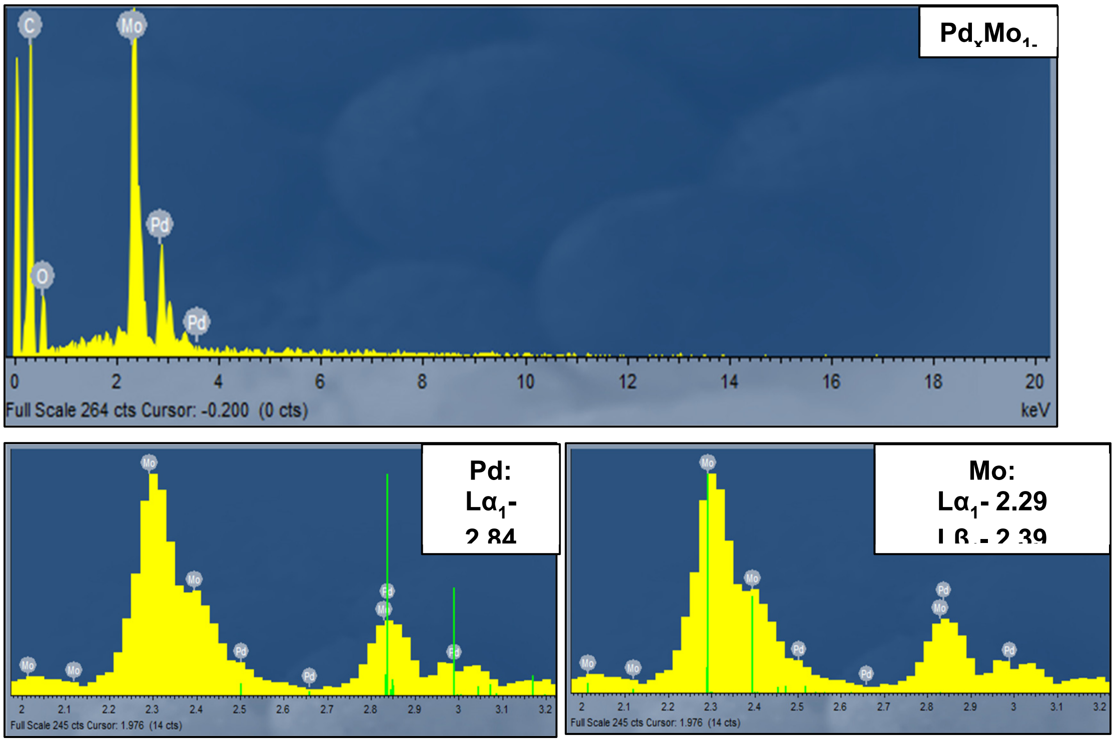

Figure S6. Example of EDS spectra of $Pd_xMo_{1-x}C$.

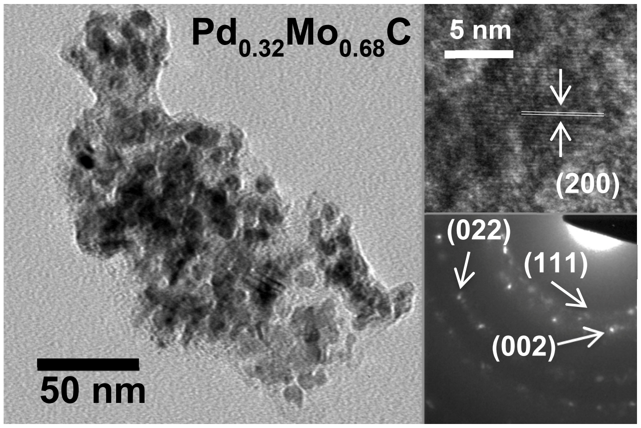

Figure S7. Bright field TEM image of Pd-Mo cubic ternary carbide (32% addition) with lattice fringe analysis and SAED.

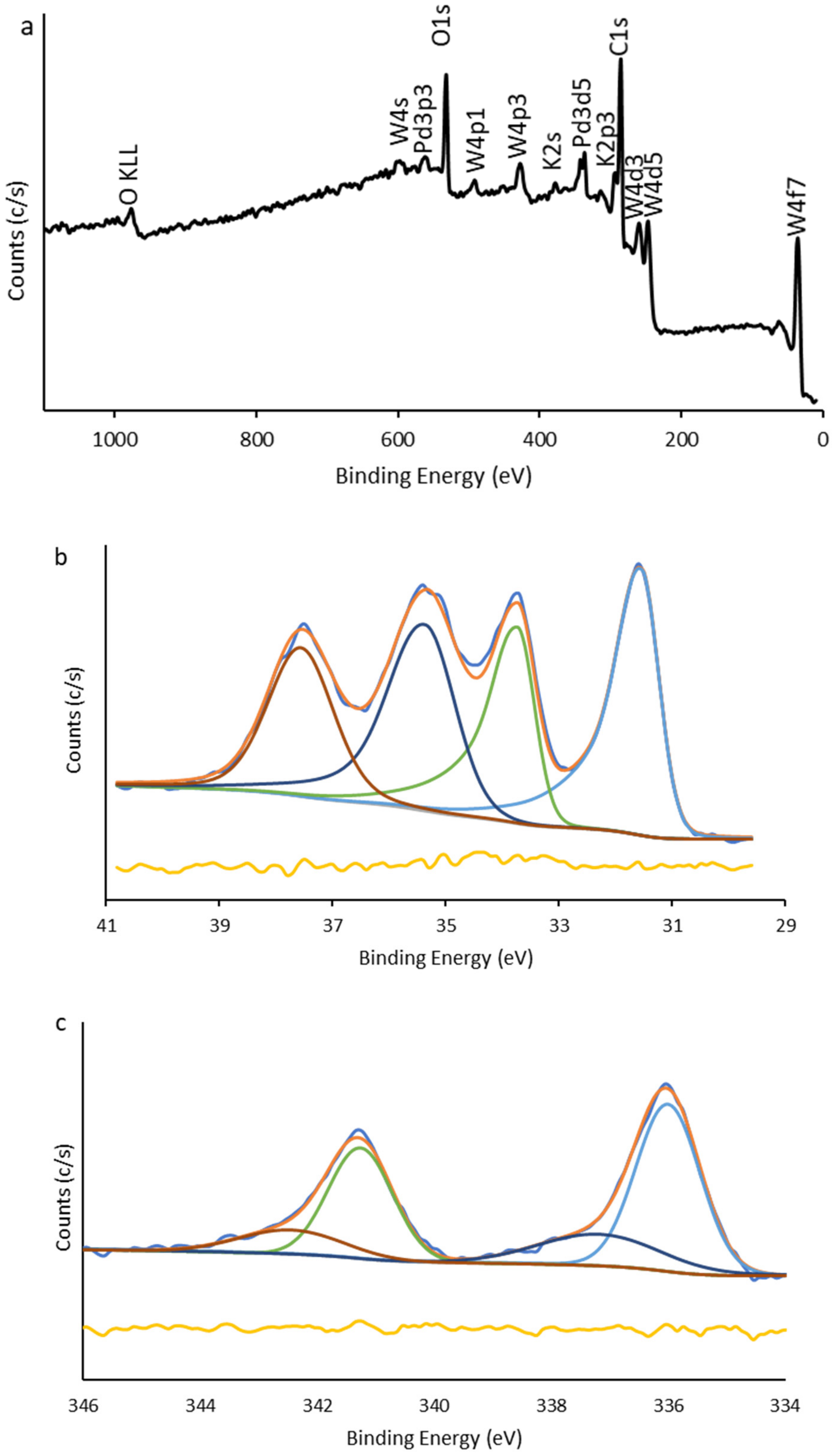

Figure S8. XPS Data for PdWC with (a) survey scan, (b) W4f region, and (c) Pd3d region.

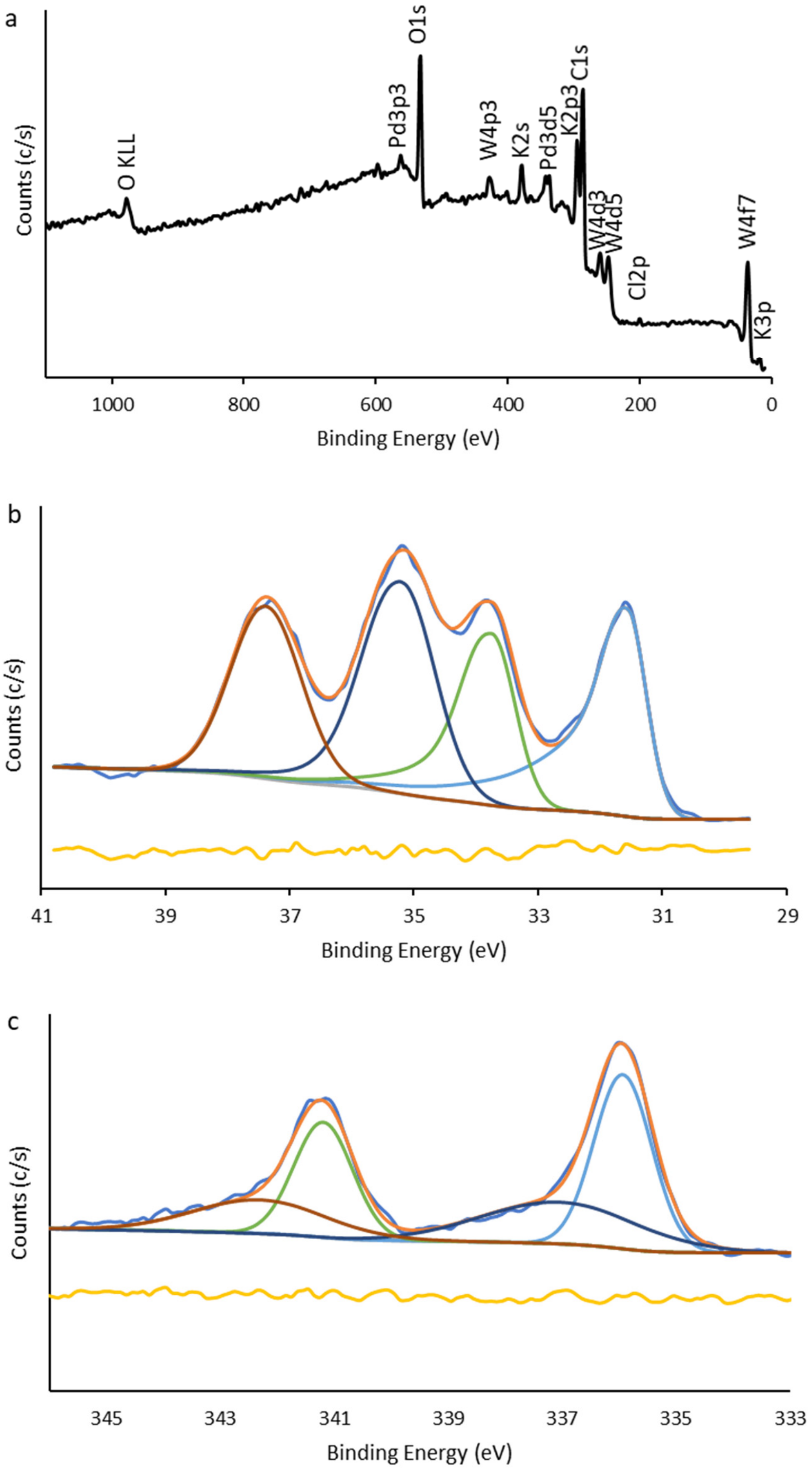

Figure S9. XPS Data for $(PdW)_2C$ with (a) survey scan, (b) W4f region, and (c) Pd3d region.

<u>Supporting info references</u>:

Kurlov, A.S.; Gusev, *A.I. Inorganic Materials* **2006** 42 (2), 121–127
